# Vertigo and Cytotoxic Lesions of the Corpus Callosum: Report with Review of the Literature

**DOI:** 10.1155/2021/5573822

**Published:** 2021-06-18

**Authors:** John Rolshoven, Katelyn Fellows, Rolando Ania, Burton J. Tabaac

**Affiliations:** ^1^University of Nevada, Reno School of Medicine, 1155 Mill Street, W-11, Reno, NV 89502, USA; ^2^Renown Institute for Neurosciences, 75 Pringle Way, Reno, NV 89502, USA; ^3^Acute Care Neurology, Renown Health, 1155 Mill St, Reno, NV 89502, USA

## Abstract

**Background:**

The term cytotoxic lesions of the corpus callosum (CLOCCs) encompasses the entity reversible splenial lesion syndrome (RESLES). RESLES typically presents with altered levels of consciousness, seizures, and delirium and is distinguished radiographically by reversible focal lesions of the splenium of the corpus callosum. This disease pathology is associated with withdrawal of antiepileptic medications, infections, metabolic disturbance, or high-altitude cerebral edema.

**Methods:**

We presented an otherwise healthy 72-year-old female that was consulted for an episode of isolated vertigo lasting four hours. Initial workup included CT head without contrast, CT angiogram head and neck, and MRI brain with and without contrast. The patient experienced recurrent episodes of vertigo at one and four months after initial presentation. An extensive workup at one month included a wide spectrum of laboratory tests and repeat imaging.

**Results:**

Noncontrast CT of the head and CT angiogram of the head and neck were reassuring. MRI brain with and without contrast demonstrated hyperintensity in the splenium of the corpus callosum on FLAIR sequencing. A follow-up visit at one month revealed vitamin B12 deficiency and unchanged hyperintensity of the splenium of the corpus callosum. History and workup were negative for typical risk factors associated with RESLES.

**Conclusion:**

An otherwise healthy patient who presented with an isolated episode of vertigo was discovered to demonstrate radiographic features consistent with RESLES but lacked the common risk factors and typical presentation of RESLES. This case expands the possible clinical presentation of RESLES and highlights the possible relationship between vitamin B12 deficiency and radiographic features of RESLES.

## 1. Introduction

Posterior Reversible Encephalopathy Syndrome, PRES, is characterized by the presence of white and gray matter changes, with a suggestion of vasogenic edema in the posterior occipital and parietal lobes, in conjunction with acute neurological symptoms [1]. Other notable areas of involvement include the cerebellum and brainstem. Radiologically, although both PRES and a cerebral infarction may affect the posterior regions, sparing of the calcarine and paramedian regions of the occipital lobe are typical with posterior cerebral infarction. Symptoms can develop acutely or subacutely and may include encephalopathy, seizures, headache, and/or visual disturbances. Hypertension is the most common precipitating factor, with other causes being sepsis, renal failure, autoimmune disorders, and cytotoxic or immunosuppressive drugs [2]. One report in the literature details a case of PRES secondary to newly diagnosed HIV prior to the initiation of antiretrovirals [3].

Reversible splenial lesion syndrome (RESLES) is considered a rare subset of PRES. RESLES is an uncommon condition that lacks a single underlying cause or classic presenting symptoms. Symptoms of RESLES are often associated with an underlying condition, with common symptoms including mildly altered states of consciousness, delirium, and seizures [4]. RESLES is characterized radiographically by T2 diffusion-weighted hyperintensity in the splenium of the corpus callosum.

Precipitating factors of RESLES may include infection, such as various forms of encephalitis, severe metabolic disturbances, withdrawal of antiepileptic medications, and high-altitude induced cerebral edema. Pathogens associated with RESLES include influenza A, mumps, varicella zoster, adenovirus, and *E.coli* [5]. The particular pathophysiology remains elusive, yet lesions of RESLES are thought to reflect intramyelinic edema, influx of inflammatory cells, macromolecules, and cytotoxic edema [6].

Like PRES, clinical presentation for RESLES can be highly variable, with common symptoms inclusive of mildly altered states of consciousness, delirium, and seizures. In some reported cases, patients with RESLES experience only nausea, vomiting and headaches [6]. While no universally agreed upon diagnostic criteria exist, T2 diffusion-weighted imaging has been shown to demonstrate rounded or ovoid hyperintensity in the splenium of the corpus callosum [7]. As seen with patients diagnosed with PRES, complete clinical and radiographic resolution occurs after weeks to months [5–7] in patients with RESLES. While most cases spontaneously resolve, patients for whom a poor prognosis is portended include those that present with acute onset of severe disturbance of consciousness, evidence of extracallosal lesions, and/or with neuroelectrographic (EEG) changes [6].

## 2. Case Presentation

A 72-year-old Caucasian woman presented with acute onset vertigo, described as “tumbling” that awoke her from sleep. This episode lasted greater than four hours, with associated nausea, moderate-intensity headache, tinnitus affecting her right ear, and with slurring of speech. Symptoms were not relieved by lying flat or remaining still. She denied numbness, weakness, or acute visual changes. Her past medical history was notable for a thirty-year history of episodic vertigo, which she describes as a “spinning,” dissimilar to the chief complaint. Prior episodes were relieved by lying flat; the patient underscored that the current episode is different in semiology. She denied past medical history of seizures, hypertension, dyslipidemia, smoking, alcohol use, or illicit drug use. She reported a family history of stroke in the aged and multiple sclerosis in several family members. She did not report any recent illness, medication changes, sick contacts, or recent travel.

Her blood pressure on arrival was 145/94, and the neurological exam was normal, including the presence of normal cranial nerves, language, reflexes (2+ throughout and symmetric), normal strength, tone, sensation, coordination, and gait. Initial serum testing reflected normal CBC and a BMP evident for mildly decreased potassium of 3.1 (reference range: 3.6–5.5 mmol/L).

**Figure 1:**
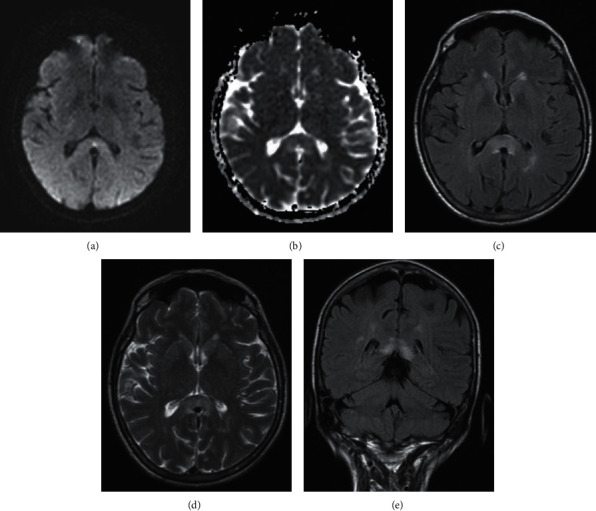
MR brain with and without contrast on admission. (a) DWI, axial cut. (b) ADC axial cut. (c) FLAIR axial cut. (d) T2 axial cut. (e) FLAIR coronal cut. Imaging reveals hyperintensity in the splenium of the corpus callosum.

**Figure 2:**
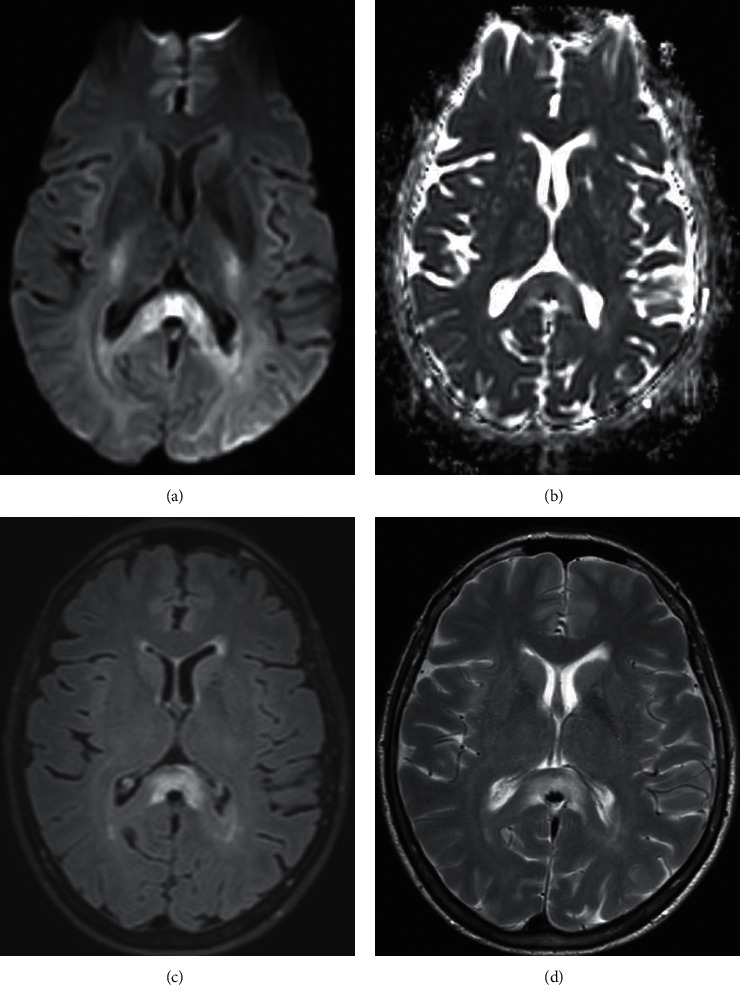
MR brain with and without contrast at one month follow up. (a) DWI, axial cut. (b) ADC axial cut. (c) FLAIR axial cut. (d) T2 axial cut. In comparison, the corpus callosum lesion is stable compared to prior imaging with the absence of novel interval lesions.

Noncontrast CT of the head and CT angiogram of the head and neck, with CT perfusion, were reassuring, apart from incidental narrowing of the right internal carotid artery, consistent with atherosclerosis, prior dissection, or congenital hypoplastic artery. The patient was admitted for additional workup and treatment. The assessment included an MRI brain with and without contrast. A punctate focus in the midline splenium corpus callosum showed hyperintensity on DWI and restricted diffusion on ADC imaging, initially believed to represent a small infarct versus foci of PRES (Figures 1(a) and 1(b)). Imaging also showed mild supratentorial white matter disease, consistent with microvascular ischemic change versus demyelination (Figures 2(a)–2(c)). Diffuse hazy T2 and FLAIR hyperintensities in the splenium of the corpus callosum were suggestive of vasogenic edema (Figures 2(a)–2(c)). The hyperintensity in the splenium of the corpus callosum was consistent with typical radiographic findings of RESLES. Vertiginous symptoms had spontaneously improved within five hours' status after onset, and she recovered to baseline within less than 24 hours. She was treated for suspected stroke and discharged on aspirin 325 mg daily and atorvastatin 40 mg nightly for secondary prevention.

In the stroke bridge outpatient clinic, one month after the initial presentation, the patient complained of recurrent bouts of intermittent vertigo, yet exhibited normotension (120/84) and a normal neurological examination. At that juncture, further workup was recommended including repeating MRI of the brain, obtaining MRI of the cervical and thoracic spine, considering a lumbar puncture to seek evidence of elevated oligoclonal bands, and obtaining additional serum studies. The patient was compliant in completing the blood work and repeat interval MRI brain only, despite encouragement to proceed with spinal tap. No significant laboratory abnormalities were noted testing of CMP, TSH, thiamine, pyridoxine, vitamin D level, compliment panel, toxicology panel, coagulation panel, serum paraneoplastic autoantibody panel, autoimmune antibodies, ESR, CRP, rheumatoid factor, syphilis, quantiferon gold, heavy metals (including arsenic, lead, cadmium, mercury, copper, and zinc), and varicella antibody. Vitamin B12 level was subtherapeutic resulting at 168 (reference range: 211–911 pg/ml), homocysteine was elevated at 16.2 (reference range: 11 umol/L), and methylmalonic acid was normal. Repeat MRI brain showed a stable corpus callosum lesion, with no novel lesions (Figures 2(a)–2(d)). MRI cervical and thoracic spine were ordered to evaluate for demyelinating disease, but not completed due to patient preference.

The findings were consistent with vitamin B12 deficiency which was treated with 1000 micrograms of cyanocobalamin IM in the clinic and 5000 micrograms of cyanocobalamin sublingual daily. Four months after initial presentation, the patient presented to the emergency department with recurrence of vertiginous symptoms, with nausea, and associated diarrhea. The episode lasted four hours and spontaneously resolved. Neurological examination was normal, and a repeat CTA head and neck showed no changes from before. An MRI brain was recommended to monitor interval improvement or resolution of a known hyperintense lesion after being treated with vitamin B12.

## 3. Discussion

In this report, we present a case highlighting the typical radiographic finding suggestive of RESLES. Our patient had a thirty-year history of severe, recurrent bouts of vertigo of varying severity. The patient lacked the typical associated risk factors of RESLES. After reassessment, the presentation and radiographic findings were attributed to vitamin B12 deficiency. As her presentation was not typical for RESLES and the lesion did not resolve within the anticipated time frame, we cannot confidently attribute this as an incidental finding, yet further workup was limited given compliance and preference for comfort over obtaining a conclusive diagnosis to attribute her symptoms. History, physical examination, laboratory studies, and imaging did not yield a clear etiology; thus, other possible entities to be considered on the differential diagnosis include demyelinating disorders, CNS vasculitis, CNS sarcoidosis, primary CNS malignancies, and subacute viral infections such as West Nile encephalitis. At the time of this report, the patient has continued to refuse additional testing.

As of the time of this report, the medical term cytotoxic lesions of the corpus callosum (CLOCCs) encompasses transient lesions of the splenium of the corpus callosum, mild encephalitis/encephalopathy with a reversible isolated SCC lesion (MERS), and reversible splenial lesions and reversible splenial lesion syndrome (RESLES). Lesions of the corpus callosum are rare and often involve the posterior section of the splenium. Clinical signs of corpus callosum lesions vary but include confusion (50–60%), ataxia (33–43%), dysarthria (13–43%), seizure (10–40%), headache (16–23%), hemiparesis (5–27%), and increased muscle tone (7–16%) [8]. Patients may also experience numbness, dysarthria, and vertigo [9]. Depending on the underlying etiology, patients can present with a range of signs and symptoms that may not be specific or purely attributable to corpus callosum lesions alone. Therefore, MR imaging is the most useful diagnostic tool in differentiating corpus callosum lesions.

Etiologies of corpus callosum lesions include vascular, traumatic, metabolic, neoplastic, demyelinating, infectious, leukodystrophy, and idiopathic causes. Isolated infarction of the corpus callosum is rare given anastomotic connections and vascular anatomy. Acute infarctions classically exhibit hyperintensity on DWI sequencing, followed by edema, and result in gliosis and atrophy. Clinical symptoms include weakness and/or numbness of the limbs, dysarthric speech, and vertigo [9]. Severe trauma may lead to diffuse axonal injury of the splenium of the corpus callosum [9]. Severe hypoglycemia can cause excitotoxic brain injury leading to cytotoxic edema, affecting the corpus callosum [10]. Severe hyponatremia may lead to cytotoxic edema of the corpus callosum. The most common tumors of the corpus callosum are CNS lymphoma and glioblastoma, which are distinct on neuroimaging as they cross the midline [11]. Demyelinating lesions seen in multiple sclerosis typically involve the corpus callosum, internal capsule, periventricular white matter, and pons. On T2-weighted MR imaging, these demyelinating lesions appear as diffuse, hyperintense, and contrast enhanced when deemed acute. Marchiafava–Bignami disease is a toxic encephalopathy marked by a degenerative process of the corpus callosum induced by vitamin B complex deficiency; in this entity, MR imaging exhibits swelling of the corpus callosum with T2 hyperintensities [11]. RESLES is a rare type of corpus callosum lesion that is differentiated by its radiographic features, since the clinical symptoms are nonspecific. It is essential to differentiate RESLES from other corpus callosum lesions given differences in treatment and prognosis.

The differential diagnosis for vertigo is broad and includes central and peripheral causes. Central etiologies of note include space-occupying lesions, brainstem ischemia, cerebellar hemorrhage, Chiari malformation, and multiple sclerosis. The clinicians involved in our presented patient's case entertained a likely association of the complained episodic vertigo with the resultant vitamin B12 deficiency, yet these may be independent findings. The patient will be counselled and encouraged to conduct repeat MR imaging for interval changes and to obtain diagnostic validity at the scheduled outpatient clinic visit.

## 4. Conclusions

This interesting case demonstrates the need for further research related to RESLES to better understand underlying etiologies that may contribute to callosal lesions. In our presented patient, none of the reported or well-known etiologies were evident, with her radiographic findings consistent with the lesions typically described and reported with RESLES. It is unknown if our patient's recurrent bouts of vertigo and vitamin B12 deficiency are related to the neuroimaging findings of isolated corpus callosal lesions as this is more so correlation rather than necessary causation in the absence of additional agreed upon testing.

## Data Availability

Readers can access the data supporting the conclusions of this case report by contacting the corresponding author at johnrolshoven@gmail.com.
